# A novel human pluripotent stem cell-based assay to predict developmental toxicity

**DOI:** 10.1007/s00204-020-02856-6

**Published:** 2020-07-22

**Authors:** Karin Lauschke, Anna Kjerstine Rosenmai, Ina Meiser, Julia Christiane Neubauer, Katharina Schmidt, Mikkel Aabech Rasmussen, Bjørn Holst, Camilla Taxvig, Jenny Katarina Emnéus, Anne Marie Vinggaard

**Affiliations:** 1 National Food Institute, Technical University of Denmark, Kemitorvet, 2800 Kongens Lyngby, Denmark; 2 Department for Biotechnology and Biomedicine, Technical University of Denmark, Produktionstorvet, 2800 Kongens Lyngby, Denmark; 3 Fraunhofer Institute for Biomedical Engineering, Joseph-von-Fraunhofer-Weg 1, 66280 Sulzbach, Germany; 4 Fraunhofer Project Center for Stem Cell Process Engineering, Neunerplatz 2, 97082 Würzburg, Germany; 5 Bioneer A/S, Kogle Allé 2, 2970 Hørsholm, Denmark

**Keywords:** Human-induced pluripotent stem cells, Embryonic stem cell test, Developmental toxicity, Thalidomide, Epoxiconazole, Valproic acid

## Abstract

There is a great need for novel in vitro methods to predict human developmental toxicity to comply with the 3R principles and to improve human safety. Human-induced pluripotent stem cells (hiPSC) are ideal for the development of such methods, because they are easy to retrieve by conversion of adult somatic cells and can differentiate into most cell types of the body. Advanced three-dimensional (3D) cultures of these cells, so-called embryoid bodies (EBs), moreover mimic the early developing embryo. We took advantage of this to develop a novel human toxicity assay to predict chemically induced developmental toxicity, which we termed the PluriBeat assay. We employed three different hiPSC lines from male and female donors and a robust microtiter plate-based method to produce EBs. We differentiated the cells into cardiomyocytes and introduced a scoring system for a quantitative readout of the assay—cardiomyocyte contractions in the EBs observed on day 7. Finally, we tested the three compounds thalidomide (2.3–36 µM), valproic acid (25–300 µM), and epoxiconazole (1.3–20 µM) on beating and size of the EBs. We were able to detect the human-specific teratogenicity of thalidomide and found the rodent toxicant epoxiconazole as more potent than thalidomide in our assay. We conclude that the PluriBeat assay is a novel method for predicting chemicals’ adverse effects on embryonic development.

**Electronic supplementary material:**

The online version of this article (10.1007/s00204-020-02856-6) contains supplementary material, which is available to authorized users.

## Introduction

Reproductive toxicity comprises all adverse effects of chemicals on the fertility and sexual function of adult males and females as well as the developmental toxicity of the offspring. Testing chemicals in vivo for reproductive toxicity according to the current regulations requires a large number of animals due to the necessary assessments of the first and, in some cases, the second generations (Van der Jagt et al. 2004). Moreover, in vivo test systems are not only expensive, time and labor-intensive, but do not always translate well into human developmental toxicity. To reduce the number of test animals and to better predict human toxicity, in vitro systems applying human cell models for a faster and more accurate prediction are urgently needed (Seiler et al. 2011; Krug et al. 2013; Leist et al. 2013; Luz and Tokar 2018).

Human embryonic development is a complex process in which numerous molecular and cellular events are orchestrated simultaneously, and which are all prone to perturbations by chemicals. Some of these processes can be mimicked in vitro as a surrogate for the developing embryo. Recent test methods have focused on neural development (Krug et al. 2013; Dreser et al. 2020), heart development (Adler et al. 2008; Seiler and Spielmann 2011), or multi-lineage differentiation (Jagtap et al. 2011; Meganathan et al. 2012; Krug et al. 2013) from mouse or human pluripotent stem cells.

It is assumed that human in vitro cell cultures should be more predictive of effects in humans; however, the widely used human embryonic stem cells (hESCs) bear ethical concerns, because human embryos are sacrificed for their retrieval (Zink et al. 2020). This can be overcome using human induced pluripotent stem cells (hiPSC), which are generated from somatic cells by adding reprogramming factors to reset their cell identity and recreate their pluripotency (Yamanaka and Takahashi 2006). HiPSC can easily be derived from individuals of different gender and ages, and have recently been used for several toxicological studies and shown to predict for example cardiac toxicity of cosmetic compounds (Chaudhari et al. 2018; Luz and Tokar 2018). Their use for predicting developmental toxicity has also been validated, as transcriptome changes during multi-lineage differentiation upon exposure to valproic acid were similar between hESCs and hiPSCs (Shinde et al. 2016).

Despite their many advantages, the use of hiPSC for developmental toxicity testing is still very rare (Luz and Tokar 2018; Zink et al. 2020). Most studies so far have compared hiPSC and hESC upon exposure to developmental toxicants to determine whether hiPSC is a valid alternative to hESCs: In an elegant study from 2011, the authors compared the transcriptomes of spontaneously differentiated EBs derived from hiPSC and hESC upon exposure to all-trans retinoic acid. By examining developmental pathways of gastrulation and organogenesis, they showed that the changes in gene expression were very similar between the two cell types (Mayshar et al. 2011). A similar trend was observed after exposure of hiPSC and hESC in monolayer to 13-cis-retinoic acid during cardiac differentiation (Liu et al. 2018) and in a transcriptome-based assay upon exposure to valproic acid (Shinde et al. 2016). Furthermore, the suitability of hiPSC to predict teratogenic effects has been shown in a 2D assay that measures the ratio of the metabolic markers ornithine and cysteine in undifferentiated hESC grown as monolayer in 96-well plates (devTOX quickPredict, devTOX^qP^) (Palmer et al. 2017).

During the last decades, however, it has become clear that monolayer culture is not optimal for many cell types, and that 3D culture supports the cells with chemical and physical cues more closely related to an in vivo environment (Benton et al. 2009; Lauschke et al. 2017). This is also true for pluripotent stem cells as they form self-organized EBs when forced into aggregates (Martin and Evans 1975). EBs are structurally and functionally similar to the blastocyst, and can undergo differentiation programs such as in the early developing embryo (Brickman and Serup 2016).

Consequently, we set out to develop an advanced assay for developmental toxicity using cardiomyocyte differentiation of hiPSC in 3D, which we term the PluriBeat assay. Importantly, we used three different hiPSC lines to investigate inter-line variability as well as male and female cells to exclude any gender-specific effects. We optimized an advanced and recently developed differentiation method for use with these three cell lines (Fischer et al. 2018), and differentiated them into beating cardiomyocytes in only 8 days. Importantly, we used the 3D culture of hiPSC in EBs and showed that these indeed undergo key developmental changes similar to early mammalian embryos. We developed an assay based on these spheroids with cardiac fate that is highly significant with respect to human embryogenesis while at the same time being robust, fast, and reproducible. Finally, we tested our assay with thalidomide, valproic acid, and the rodent toxicant epoxiconazole.

## Methods

### hiPSC culture

Three hiPSC lines were used: BIONi010-C (Bioneer A/S, Hoersholm, Denmark), IMR90-1, and IMR90-4 (WiCell, Madison, USA). hiPSC were cultured in mTeSR™1 serum-free medium (STEMCELL Technologies, Vancouver, Canada) on hESC-Qualified Matrigel (354,277, Corning, Corning, USA) coated cell culture dishes (Nunclon™ Delta Surface, Thermo Fisher Scientific, Waltham, USA). Medium was exchanged daily and morphology of the hiPSC colonies assessed. Cells were split once a week to maintain exponential growth. Cells were split by removing medium from the culture dish, washing with DPBS (Dulbecco’s Phosphate Buffered Saline, Sigma-Aldrich, St. Louis, USA), and adding 0.02% EDTA in DPBS until single cells appeared at the borders of the colonies after 1–3 min. EDTA was removed, cells suspended into small clumps in mTeSR™1 and transferred to a new microtiter plate. hiPSC were incubated at 37 °C and 5% CO_2_.

### hiPSC differentiation into cardiomyocytes

Cardiac differentiation was performed as published (Fischer et al. 2018) with slight modifications. Briefly, hiPSC were harvested from culture dishes with good colony morphology (day 5–6 after split) by removing medium and adding Gibco™ TrypLE™ Select (Thermo Fisher Scientific) for 1–2 min. As soon as single cells appeared at the borders of the colonies, TrypLE was removed by suction and the cells harvested as single cells in mTeSR-ROCK (mTeSR1 with 10 µM Y27632 dihydrochloride). Cells were counted and 5 × 10^4^ cells/ml were seeded at 100 µl per well into a 96-well Polystyrene Conical Bottom MicroWell™ Plate (249,952, Thermo Fisher Scientific). The plates were centrifuged at 500*g* for 5 min at room temperature. After 20 h of incubation, EBs had formed (one EB per well) and cardiac differentiation was induced by exchanging 80 µl medium to D0 medium. All medium recipes are included in supplementary Table 1. After 24 ± 2 h, 80 µl medium was exchanged to TS medium. After 24 ± 2 h, 80 µl medium were exchanged to Wnt medium. After 24 ± 2 h, 80 µl medium was exchanged with TS medium. Three days later (72 ± 2 h), 60 µl medium was exchanged with 80 µl fresh TS medium added per well. The following day beating of the cardiomyocyte containing EBs was scored.

### Gene expression analysis

Cells were harvested on the days indicated on the respective figures and RNA extracted with the Qiagen RNeasy Micro Kit (Qiagen, Hilden, Germany) according to the manufacturer’s instructions. RNA concentration was measured on a NanoDrop (Thermo Fisher Scientific) and 500 µg reverse transcribed into cDNA using the Omniscript^®^ Reverse Transcription Kit (Qiagen). For quantitative RT-PCR, 3.75 ng cDNA was used per well in a 384-well microtiter plate. RT-PCR was performed with the TaqMan Assay Kit (Thermo Fisher Scientific) on a QuantStudio 7 Flex (Applied Biosystems, Foster City, USA), for primers (see supplementary Table 2). Each sample was measured in technical duplicates, samples with a cycle threshold (CT) difference > 1 between duplicates were excluded (for samples with CT values < 30 only). Samples with CT values > 35 were regarded as non-detectable. Relative gene expression levels were calculated with the ∆∆CT method, and normalized to the average of the two house-keeping genes, GAPDH (Glyceraldehyde 3-phosphate dehydrogenase) and ACTB (β-actin). Expression of the house-keeping genes was monitored to be constant between the samples to allow the comparison of gene expression levels.

### Immunocytochemistry

After each step in the following protocol, reagents were removed and EBs washed three times in 500 µl PBS. On day 7 of differentiation, EBs were fixed in formalin for 20 min at room temperature. EBs were permeabilized with 500 µl 0.1% (v/v) Triton X-100 (in PBS) for 1 h at room temperature on a rotating wheel at 10 rpm. EBs were blocked in 500 µl 3% BSA (in PBS) for 1 h at room temperature on a rotating wheel at 10 rpm. Primary antibodies were diluted in 3% BSA (in PBS) and incubated with the EBs overnight at 4 °C on a rotating wheel at 10 rpm. EBs were incubated in secondary antibodies and diluted in 3% BSA (in PBS) for 1 h at room temperature in the dark on a rotating wheel at 10 rpm. Nuclear DNA was stained with DAPI (1:1000 in PBS), 500 µl per tube for 10 min at room temperature in the dark. For mounting, the EBs were transferred to glass slides with cavities (Hounisen Laboratorieudstyr, Skanderborg, Denmark), PBS was carefully removed and EBs mounted in ProLong Gold Antifade Mountant (Thermo Fisher Scientific) under a cover slip. The following primary antibodies were used: Anti-Cardiac Troponin T 1:400 (ab45932, Abcam, Cambridge, UK) and anti-Nkx2.5 1:50 (sc-376565, Santa Cruz Biotechnology, Dallas, USA). The following secondary antibodies were used: anti-rabbit AlexaFluor-568 1:500 (Molecular Probes, Eugene, USA) and anti-mouse AlexaFluor-488 1:500 (Molecular Probes). Microscopy images were taken on a Zeiss LSM710 confocal microscope.

### Resazurin assay

To determine growth in cell number of EBs during differentiation, a full 96-well plate of EBs on day 0 and day 7 of differentiation was used. For this, 60 µl/well old medium was removed and replaced by 60 µl/well fresh TS medium (supplementary Table 1). Subsequently, 100 µl/well resazurin solution (0.01 mg/ml in PBS) was added and the plates incubated at 37 °C and 5% CO_2_ for 2 h. Then, the content of each well was transferred to a black microtiter plate and fluorescence measured (EnSpire, Perkin Elmer). For blank measurements, 100 µl/well resazurin solution was added to 100 µl/well KO-DMEM and processed as samples with EBs.

### Testing of compounds

Stock solutions of thalidomide, valproic acid, and epoxiconazole were prepared at 200 mM, 600 mM, and 600 mM, respectively. The valproic acid solution was prepared in ethanol whereas thalidomide and epoxiconazole solutions were prepared in dimethyl sulfoxide (DMSO). Test compound solubility was assured by visual examination for precipitation.

Before initiating tests on cardiomyocyte differentiation for test chemicals, cell viability was determined to ascertain that any further testing was done at non-cytotoxic concentrations. On the day of the experiment, 50 µl/well hESC-Qualified Matrigel (354,277, Corning) was added to flat-bottom 96-well plates (Corning) and incubated at 37 °C and 95% humidity until use. BIONi010-C hiPSC were harvested from a 6 cm^2^ dish by Gibco™ TrypLE™ Select (Thermo Fisher Scientific) and a single-cell suspension was prepared in mTeSR™1 serum-free medium (STEMCELL Technologies). Matrigel was removed from plates and a final concentration of 1 × 10^4^ cells/well was added. After 24 h, mTESR1 medium was exchanged for fresh medium. Cell were exposed to the test chemicals after another 24 h and for 48 h in total with a vehicle kept constant across the plates at 0.1%. Thereafter, the experiment was terminated and the CellTiter-Glo^®^ 2.0 Cell Viability Assay (Promega) performed as described by the manufacturer. Assay reagent (100 µl/well) was added, plates were left on a shaker table for 2 min and successively for 10 min at room temperature. Lysates were transferred to white plates and luminescence was measured (EnSpire, Perkin Elmer). Three independent experiments were conducted with each treatment in 6–12 replicates with thalidomide tested at 25, 50, 100, and 200 µM, valproic acid at 50, 100, 200, and 400 µM, and epoxiconazole at 12.5, 25, 50, and 100 µM.

Means from independent experiments were pooled and exposure concentrations leading to more than 20% decreased cell viability was perceived as cytotoxic.

For analysis of cardiomyocyte differentiation, test compounds were tested at non-cytotoxic concentrations of 2.3, 4.5, 9, 18, and 36 µM thalidomide, 25, 50, 100, 200, and 300 µM valproic acid and 1.3, 2.5, 5, 10, and 20 µM epoxiconazole. Test compounds were added to the specific daily differentiation medium before medium exchange on the 96-well plates. Vehicle was kept constant across the plates at 0.1%. Thirty-two EBs were exposed to each concentration in each experiment. On the last day of the assay, the beat score was determined as follows: Each EB was observed until contraction was seen, though for a maximum of 10 s. If no movement was seen, the beat score 0 was given. If the entire area of the sphere contracted, a beat score of 2 was given. Everything in between was given a score of 1.

For analysis of the EB size, the EBs were imaged with a light microscope after assessing the beat score. The plates were left at room temperature to decrease the beat rate before imaging, thereby decreasing the risk of false small outliers due to contractions. The images were further analyzed with ImageJ, where the diameter of the individual EBs was measured manually. From this, the volume of each EB was calculated and the average of the controls on each plate set to 100% to calculate the volume (%) for the exposed EBs. The average of 32 EBs per condition was analyzed.

### Data processing

The beat score for each condition was determined as the average beat score of the 32 EBs. All experiments were performed in biological triplicates if not stated otherwise. For gene expression analysis and compound testing, data from 32 EBs (= 32 wells each containing one EB) were analyzed for a given concentration or time point.

The following statistical analysis was performed in GraphPad Prism (version 7): To analyze whether the gene expression differed significantly between the three cell lines, we performed a two-way ANOVA without matching and as follow-up the Tukey test. To analyze whether the CHIR99021 concentration had a significant impact on the number of beating and non-beating EBs, we performed the Fisher’s exact test. EB size was analysed using a one-way ANOVA without matching and multiple comparisons with Dunnett’s correction.

IC_25_ values were defined as absolute IC_25_ values giving the concentration that inhibits the beat score by 25%. They were calculated in Graphpad Prism (version 8) using a four-parameter logistic curve fit with the lower limit constrained to 0 and upper limit constrained to 2, as well as the parameter F set to 75 to get the absolute inhibition of 25%. Calculations were based on the beat score of > 90 EBs from three independent experiments per cell line.

The following analysis was performed in the statistical program R-studio: To analyze the impact of the test compounds on the beat score, we performed an ordinal logistic regression using the polr package in R with beat score and concentration as factors on *n* ≥ 90 EBs from three biological replicates. The LOEC was defined as the lowest observable effect concentration of test compound causing an effect with a *p* value < 0.05.

*p* values of *p* < 0.05 are indicated with ‘*’, *p* < 0.01 with ‘**’, and *p* < 0.001 with ‘***’ on the figures.

## Results

### Design of the PluriBeat assay

**Fig. 1:**
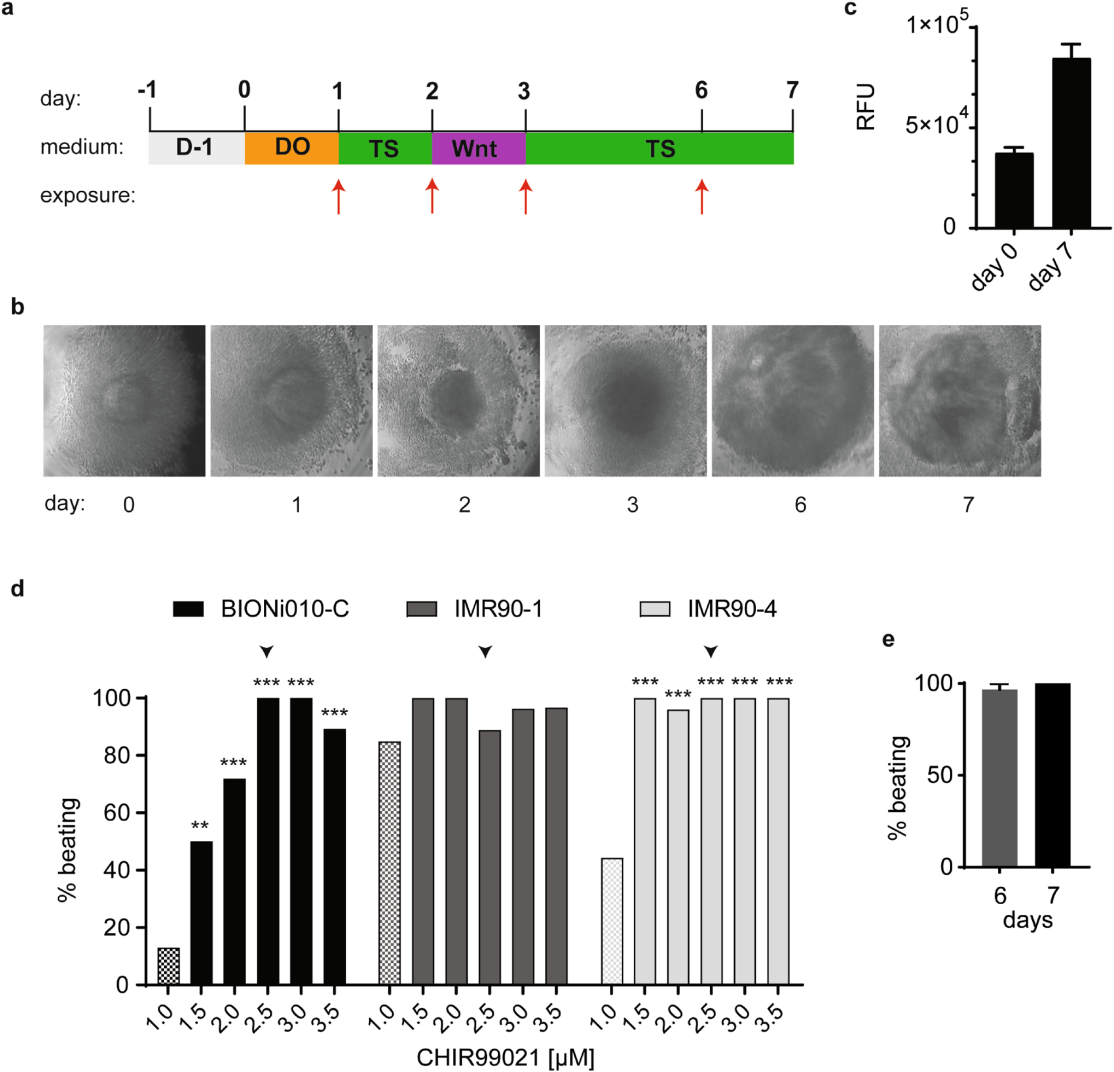
Assay design. **a** Cardiac differentiation and exposure scheme of the assay. Cells are seeded on day − 1 in mTeSR-ROCK medium (D−1) and embryoid bodies (EBs) form overnight. On day 0, medium is exchanged to day 0 medium (D0). On day 1, medium is exchanged to TS medium. On day 2, medium is exchanged to Wnt medium (Wnt). On days 3 and 6, medium is exchanged to TS medium. For exposure on the days indicated by red arrowheads, the chemicals are diluted in the respective media. **b** Light microscopy images of representative EBs with the BIONi010-C line in a well of a 96-well plate throughout differentiation (× 4 magnification). **c** Number of viable cells in the EBs on day 0 and day 7 of cardiac differentiation with the BIONi010-C line. Viable cells measured with resazurin, fluorescence measured in relative fluorescent units (RFU). Mean and SD of > 90 wells of a 96-well plate. **d** Optimizing the CHIR concentration in D0 medium, the standard concentration of 1 µM CHIR is indicated with halftoning and the optimized concentration of 2.5 µM with an arrowhead. BIONi010-C, IMR90-1, and IMR90-4 were differentiated with increasing CHIR99021 concentrations. Beating EBs were counted on day 7, one biological experiment with 14–32 EBs per condition. Statistical significance for each CHIR concentration relative to 1 µM CHIR. **e** The number of beating EBs was counted on days 6 and 7 using the BIONi010-C line, and 32 EBs were counted per experiment, mean, and SEM of three experiments

To develop the PluriBeat assay based on differentiation of hiPSC, we first set out to optimize the cardiac differentiation of hiPSC in 3D cultures. These cells have shown high variability between cell lines and, therefore, more than one cell line has been suggested to be used (Shi et al. 2016). We used the cell line BIONi010-C, which has been derived from an adult male donor (15–19 years of age) (Rasmussen et al. 2014), as well as IMR90-1 and IMR90-4, which originate from lung fibroblasts of a 10-week-old aborted human female embryo (Yu et al. 2007). With this, we obtained a set of male and female cell lines of different donor ages. We implemented a 3D protocol for cardiomyocyte differentiation to retrieve spheres with contracting cardiomyocytes as a simple readout in conical bottom 96-well plates (Zhang et al. 2015; Fischer et al. 2018). An outline of the assay is depicted in Fig. 1a. The EBs proliferated and changed in morphology throughout differentiation as representatively shown for BIONi010-C in Fig. 1b. To assess the growth of the EBs, we measured the number of viable cells in each well using resazurin on day 0 and day 7 of differentiation. This confirmed that the EBs markedly increased in cell number and that the cell number varied only little within one plate (CV 7.4% on day 0 and 8.1% on day 7) (Fig. 1c). We optimized the concentration of CHIR99021 for our three cell lines, because the optimal level of this factor is known to be cell line dependent. We tested concentrations from 1 µM (Fischer et al. 2018) to 3.5 µM, and chose 2.5 µM as the lowest concentration with significantly improved differentiation efficiency in all three cell lines (Fig. 1d). Furthermore, we counted the number of beating EBs on day 6 and day 7 of differentiation. First contractions were seen on day 6; however, robust and strong contractions of all EBs were first evident on day 7 (Fig. 1e), and hence, we set the assay readout to day 7. With this, we implemented a simple method to reproducibly create beating EBs within 7 days of differentiation in a 96-well format.

For the exposure protocol, we chose to initiate exposure on day 1 through day 6 (Fig. 1a). Moreover, we introduced a quantitative score of the readout, because exposure to chemicals impaired the beating of the EBs to varying degrees: a fully beating EB was given the value 2, an EB with impaired or only partial beat 1, and an EB without contractions was counted as 0. Examples of the three categories of beating are given in suppl. videos 1–3. From this, we calculated the average beat score for each exposure.

### Molecular characterization of the PluriBeat assay system

**Fig. 2:**
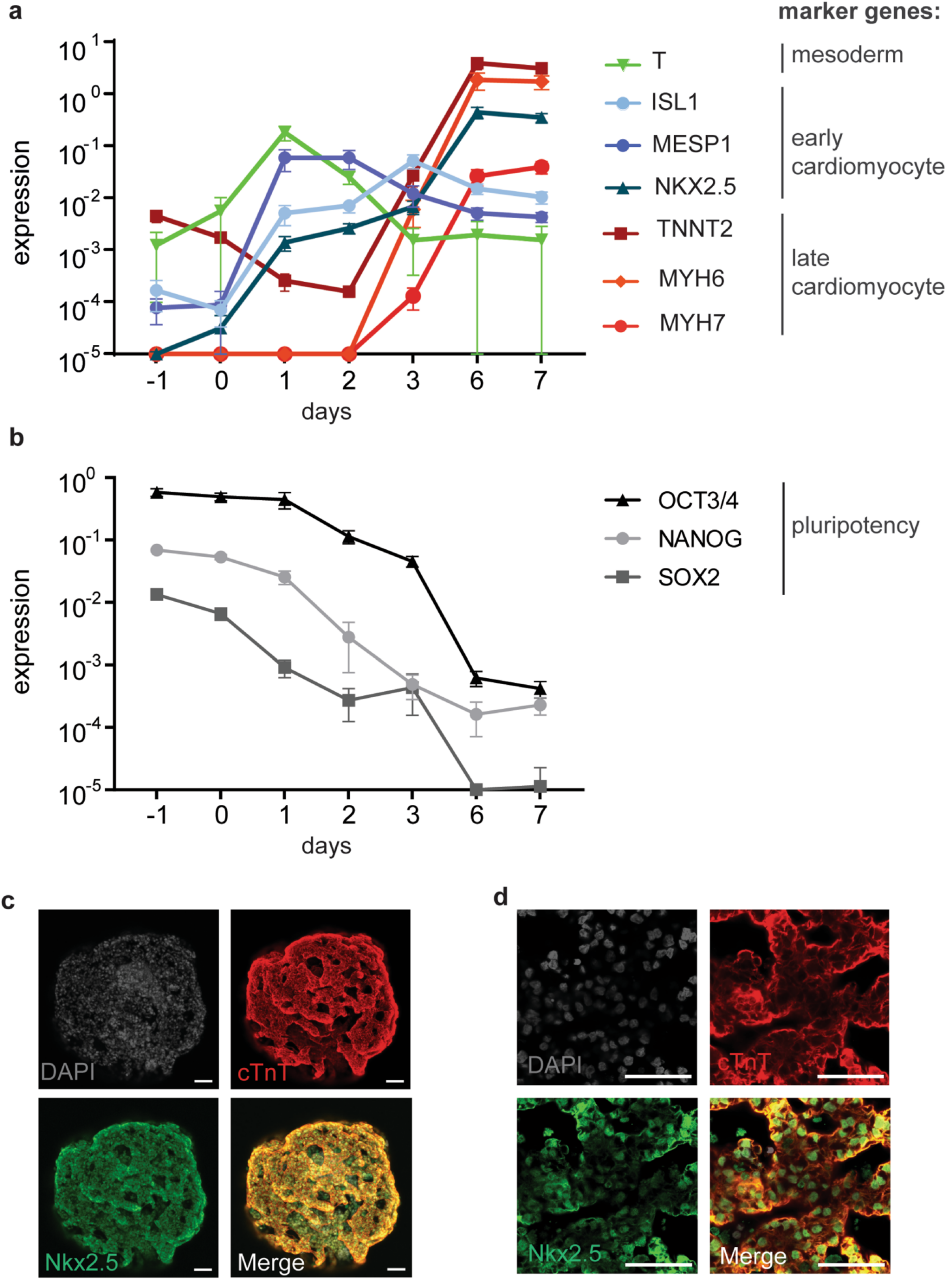
Molecular characterization. **a** Gene expression analysis of BIONi010-C during the course of differentiation. Shown are the mesoderm marker T, early cardiac marker genes (ISL1, MESP1, and NKX2.5), and late cardiac marker genes (TNNT2, MYH6, and MYH7). Mean and SEM of four experiments with BIONi010-C, expression relative to house-keeping genes. **b** Gene expression analysis of pluripotency genes during cardiac development. Mean and SEM of four experiments with BIONi010-C, expression relative to house-keeping genes. **c** Immunofluorescence analysis of EBs on day 7 of differentiation with BIONi010-C. Maximum intensity projection of a z-stack of an entire EB. Scale bar 50 µm. Shown are representative EBs of three independent differentiation experiments with > 3 EBs imaged per experiment. **d** Higher magnification of EBs as in **c**, scale bar 50 µm. Shown are representative EBs of three independent differentiation experiments with > 3 EBs imaged per experiment

It was important to analyze whether the differentiating EBs truly follow key developmental stages of the developing embryo, i.e., formation of mesoderm, followed by cardiac mesoderm, cardiac progenitors, and, finally, cardiomyocytes. To characterize this in detail, we performed qRT-PCR on the BIONi010-C hiPSC line during differentiation with a set of markers including the T gene as a mesoderm marker, the cardiac progenitor markers MESP1 and ISL1, the cardiac lineage marker NKX2.5, as well as the cardiomyocyte markers TNNT2, MYH6, and MYH7. The T gene was induced on day 0 and peaked on day 1 (Fig. 2a), confirming mesoderm formation. Expression of ISL1 and MESP1 on days 1–3 confirmed the presence of cardiac progenitor cells. Cardiac lineage marker NKX2.5 was expressed from day 1 throughout differentiation at continuously increasing levels. Cardiomyocyte markers TNNT2, MYH6, and MYH7 were markedly expressed on days 6 and 7, indicating the differentiation into cardiomyocytes (Fig. 2a). We also found that the pluripotency genes NANOG, OCT3/4, and SOX2 were efficiently downregulated during differentiation (Fig. 2b). These data together confirmed our findings that the EBs differentiated into spheroids containing beating cardiomyocytes through developmental stages that also occur in the human developing embryo.

To get a better understanding of the distribution of cardiac cells within the spheres, we investigated cardiac marker proteins across single EBs using immunofluorescence staining of Nkx2.5 and cTnT (the protein of the TNNT2 gene). As shown in Fig. 2c, both marker proteins were detected across the entire EB, confirming the presence of cardiomyocytes. Furthermore, cTnT was detected mostly in the cytoplasm and connecting neighboring cells, consistent with its function in the sarcomere complex of cardiomyocytes. Nkx2.5 was mainly detected in the nucleus of these cells, which was expected, since it acts in the nucleus as a cardiac transcription factor (Fig. 2d). We, therefore, concluded that our protocol ensures a robust and reproducible differentiation into cardiomyocytes.

### Characterization of three different hiPSC lines

**Fig. 3:**
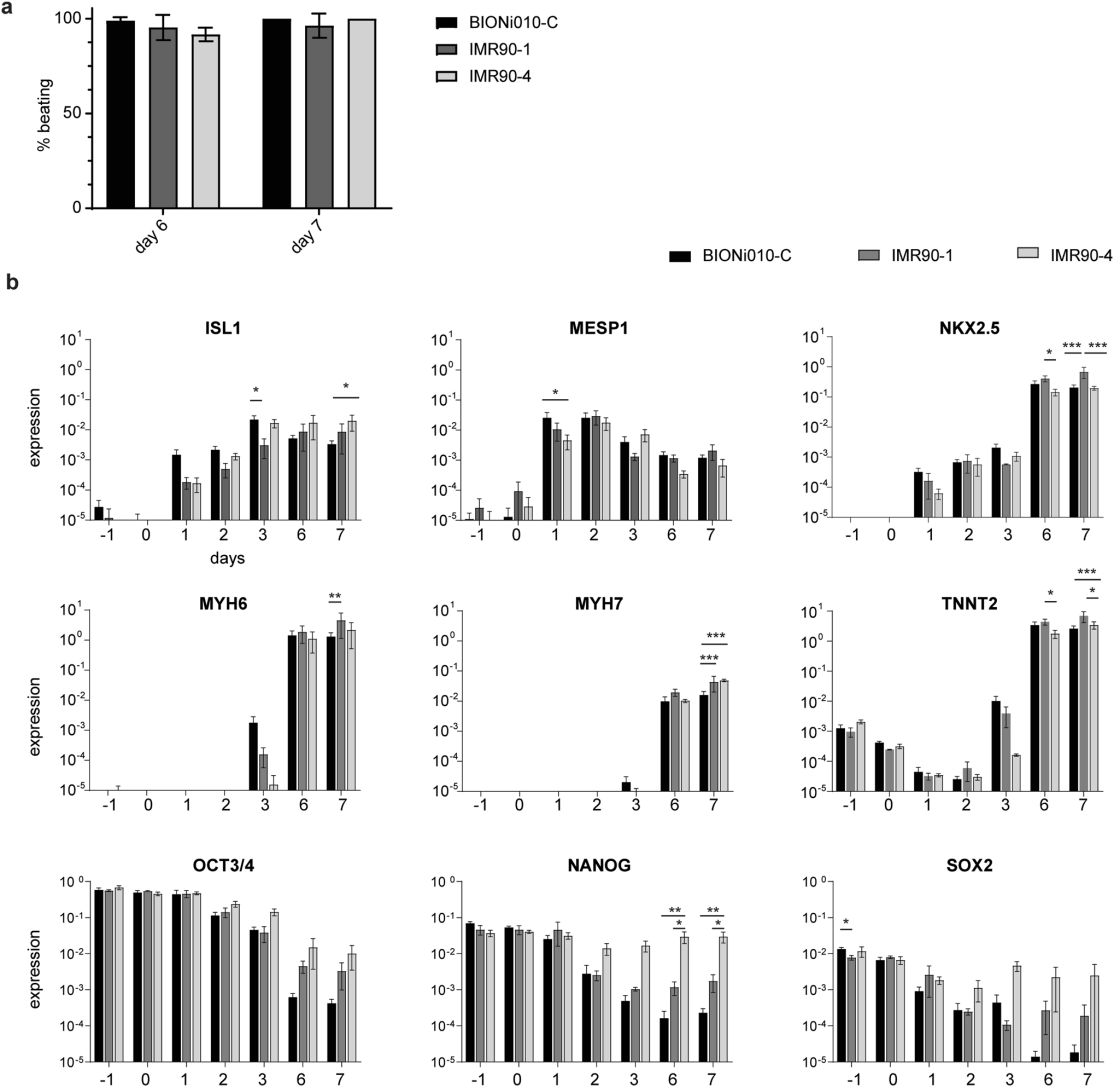
Differentiation efficiency and gene expression analysis for three cell lines. **a** All three cell lines (BIONi010-C, IMR90-1, and IMR90-4) were differentiated into cardiomyocytes and beating embryoid bodies (EBs) counted on day 6 and day 7. Thirty-two EBs were counted per experiment. Shown in percent of all counted EBs, mean and SEM of three differentiation experiments with each cell line. **b** All three cell lines (BIONi010-C, IMR90-1, and IMR90-4) were differentiated into cardiomyocytes and samples taken every day of differentiation. Mean and SEM of ≥ 2 differentiation experiments for each cell line. Stars indicate significant differences between cell lines calculated with a two-way ANOVA. Columns without stars do not differ significantly. *n* = 4 (BIONi010-C), *n* = 2 (IMR90-1 and IMR90-4)

Next, we characterized cardiomyocyte differentiation efficiency with three different hiPSC lines BIONi010-C, IMR90-1, and IMR90-4, and first counted the number of beating EBs on day 6 and day 7 of differentiation. On day 6, not all EBs contracted robustly in all three cell lines, with BIONi010-C showing the highest number of beating EBs and IMR90-4 the lowest number. However, on day 7, the BIONi010-C and IMR90-4 cell lines gave rise to 100% beating EBs. In IMR90-1 cells, the efficiency did not reach 100% in all experiments, but with an average of more than 95% beating EBs, the performance was considered acceptable (Fig. 3a).

We also examined whether the cell lines differentiated in a similar way and, therefore, performed qRT-PCR with early and late cardiac markers in all three cell lines. We found approximately the same quantitative expression of marker genes in all three cell lines, with early markers being upregulated before the late markers (Fig. 3b). There was almost no statistically significant difference in marker gene expression between the cell lines, and the most marked differences were for NKX2.5, MYH7, and TNNT2 on day 7 (Fig. 3b). We also investigated the pluripotency genes NANOG, OCT3/4, and SOX2 in all three cell lines, and found that they were efficiently downregulated during differentiation. However, IMR90-4 repeatedly showed the smallest decrease in expression of the three pluripotency genes (Fig. 3b), which was significant for NANOG on days 6 and 7.

Overall, these results illustrate that the origin of the cell lines had little influence on the expression of cardiac marker genes during differentiation. Together with our results of almost 100% beating cardiac EBs in all three cell lines, we concluded that the differentiation process is reproducible between the three lines used in this study.

### Testing thalidomide, valproic acid, and epoxiconazole in the three cell lines

**Fig. 4:**
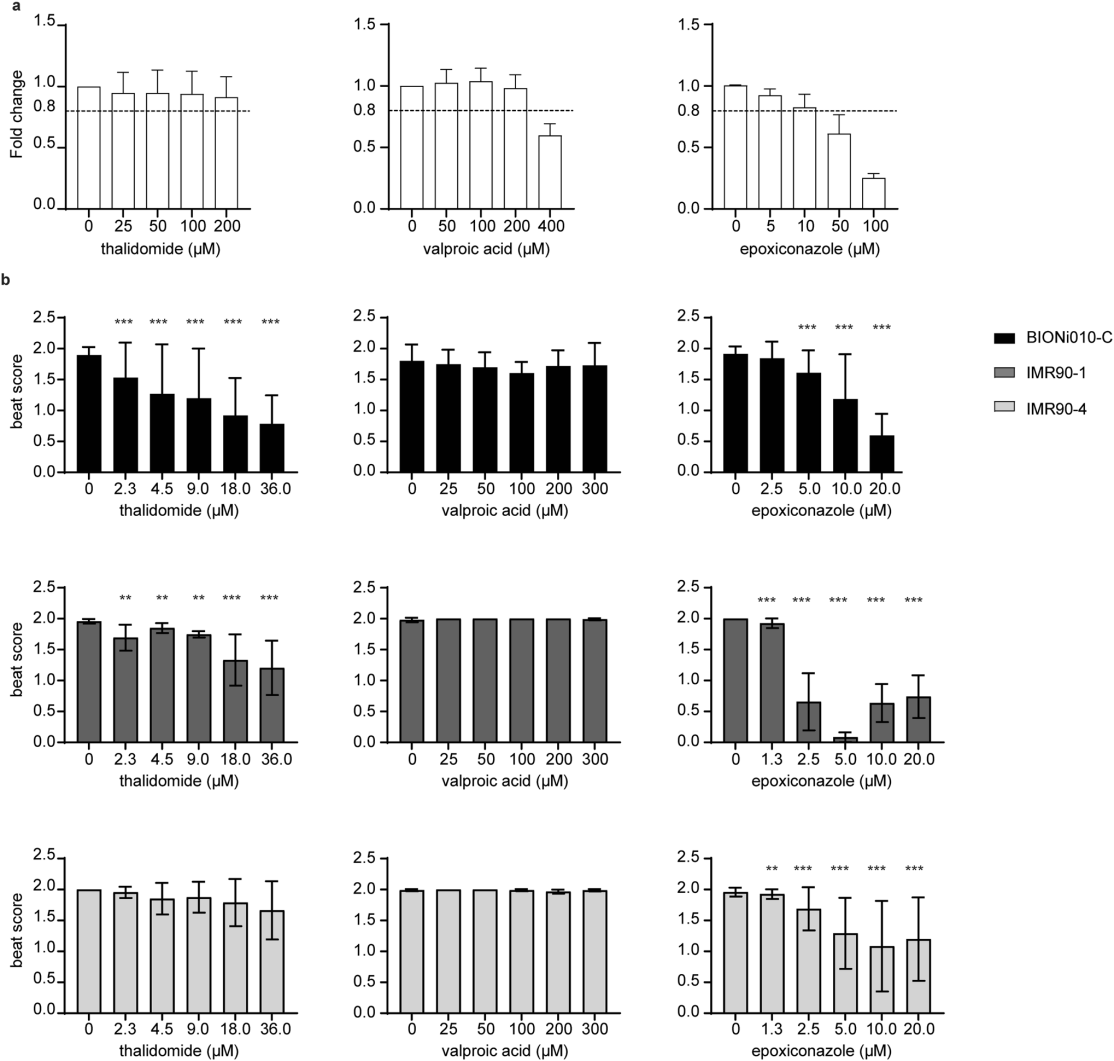
Testing thalidomide, valproic acid, and epoxiconazole in the PluriBeat assay. **a** Cytotoxicity of test compounds. BIONi010-C were grown in monolayer and exposed to the three reference compounds for 2 days, and then, the proportion of viable cells was assessed using an ATP based assay. Dashed line at 0.8-fold change indicates cut-off for cytotoxicity. Mean and SD of three experiments. **b** EBs made from the three cell lines BIONi010-C, IMR90-1, and IMR90-4 were exposed to increasing concentrations of thalidomide, valproic acid, or epoxiconazole, and the beat score assessed on the last day of the assay. Thirty-two EBs per condition, mean, and SD for three biological replicates of each cell line. Stars indicate significant differences of respective concentrations vs. control calculated with an ordinal logistic regression on *n* ≥ 90 EBs

To illustrate the applicability of our assay, we tested three known teratogens: first, thalidomide, which is a strong human-specific teratogen that cannot be detected in animal experiments with mice (Newman et al. 1993). According to our knowledge, its teratogenic potential has not been published with mouse stem cell-based assays. Second, we tested valproic acid as a weak teratogen (Genschow et al. 2002) and, finally, epoxiconazole, which has previously been shown to affect fetal development in rats and to be weakly embryotoxic in the mouse embryonic stem cell test (mEST) (Taxvig et al. 2007; Dreisig et al. 2013). First, we assessed the non-cytotoxic range of the compounds by measurement of ATP as a surrogate for viable cells. Thalidomide did not reduce viability in any of the concentrations tested (Fig. 4a), and we chose the concentrations 2.3, 4.5, 9, 18, and 36 µM for further testing on cardiomyocyte differentiation. VPA reduced viability by more than 20% at 400 µM (Fig. 4a), and we chose to expose EBs to 25, 50, 100, 200, and 300 µM. Epoxiconazole showed cytotoxicity from 50 µM and upward (Fig. 4a), and we, therefore, tested 1.3, 2.5, 5, 10, and 20 µM on the EBs.

Thalidomide exposure led to a concentration-dependent decline in beat score, with BIONi010-C as the cell line with the strongest response (Fig. 4b) and IMR90-4 only showing a non-significant trend. Valproic acid did not lead to a significant decrease in beat score in any of the cell lines at the maximum concentration tested (Fig. 4b). However, we cannot exclude that effects might happen at higher test concentrations. Epoxiconazole led to decreased beat scores in the three cell lines with the strongest effects observed in IMR90-1 cells (Fig. 4b). The LOEC for thalidomide in the BIONi010-C and IMR90-1 cell lines was 2.3 µM, while no LOEC could be determined for IMR90-4 (Table 1). Epoxiconazole exposure led to an LOEC of 5 µM in BIONi010-C cells, while in IMR90-1 and IMR90-4, a reduction was already seen at lower concentrations. We, therefore, also included the exposure concentration of 1.3 µM in these two cell lines. Intriguingly, we observed that the EBs at 1.3 µM were beating at a markedly increased beat rate (suppl. video 4) compared to control (suppl. video 5), before they stopped beating at 2.5 µM. We also calculated the absolute IC_25_ values, i.e., the concentrations that decreased the beat score by 25% for thalidomide and epoxiconazole (Suppl. Figure 1). Thalidomide gave a lower IC_25_ in BIONi010-C (2.0 µM) than in IMR90-1 (14.3 µM), while epoxiconazole gave a higher IC_25_ in BIONi010-C (5.2 µM in BIONi010-C vs. 1.8 µM in IMR90-1). In the IMR90-4 cells, epoxiconazole gave an IC_25_ value of 2.7 µM. (Table 1).

**Table 1:** LOEC (µM) and absolute inhibition concentration of 25% (IC_25_) with 95% confidence intervals (µM) for test compounds in three different hiPSC lines

	BIONi010-C		IMR90-1		IMR90-4	
	LOEC	IC_25_	LOEC	IC_25_	LOEC	IC_25_
Thalidomide	2.3 µM	2.0 (1.0–3.1) µM	2.3 µM	14.3 (11.3–17.8) µM	> 36 µM	> 36 µM
Valproic acid	> 300 µM	> 300 µM	> 300 µM	> 300 µM	> 300 µM	> 300 µM
Epoxiconazole	5 µM	5.2 (4.4–6.1) µM	1.3 µM	1.8 (1.6–?)* µM	1.3 µM	2.7 (1.7–3.8) µM

*No upper limit of the 95% confidence interval can be calculated > 300 µM and > 36 µM which indicate that these are the maximum concentrations tested and that we cannot exclude effects at higher concentrations

**Fig. 5:**
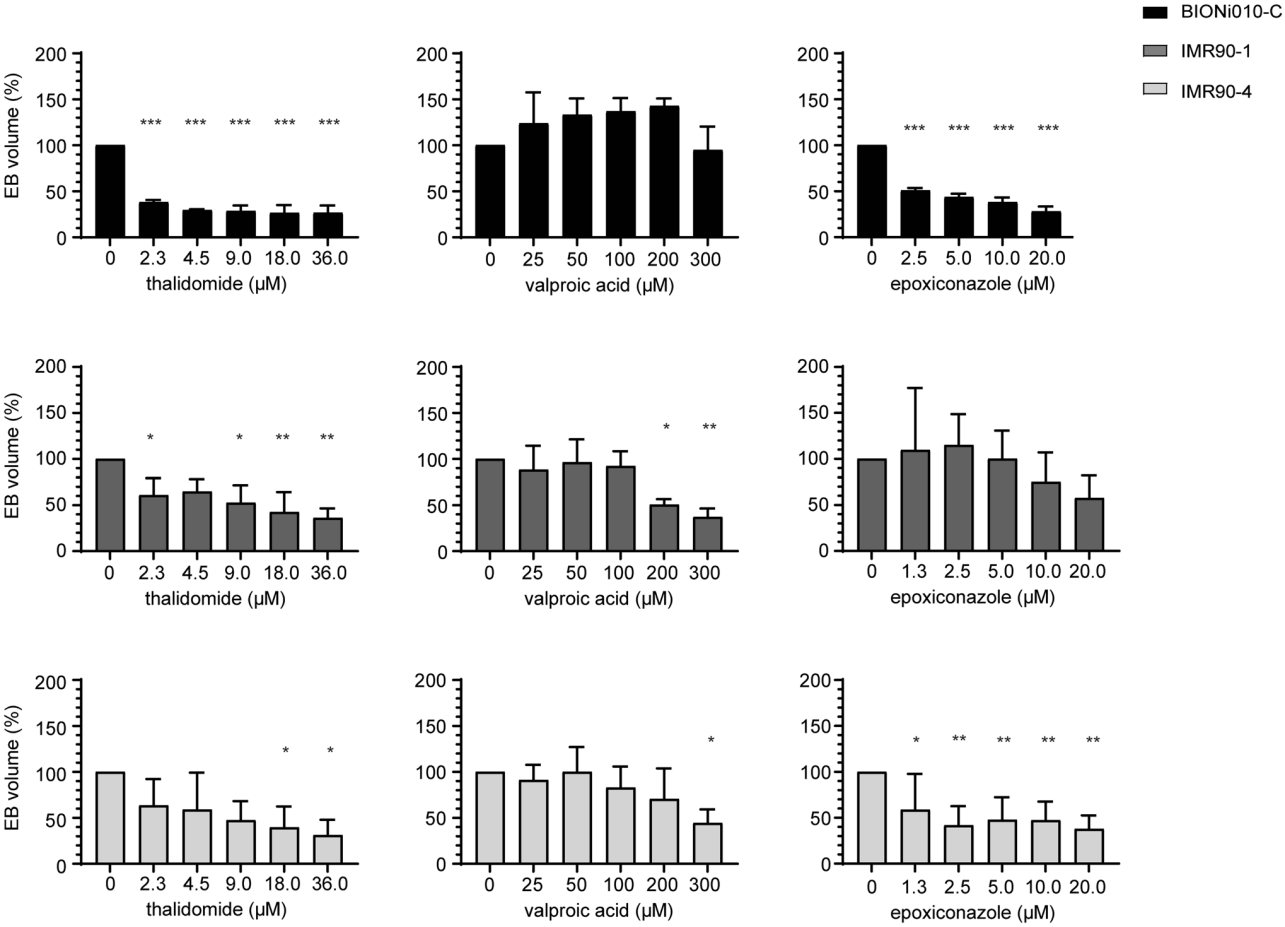
Decrease in EB volume after exposure to thalidomide, valproic acid, and epoxiconazole. EBs made from the three cell lines BIONi010-C, IMR90-1, and IMR90-4 were exposed to increasing concentrations of thalidomide, valproic acid, or epoxiconazole, and light microscopy images of each individual EB were taken on the last day of the assay. The diameter was measured to calculate the volume of the EBs, and the volumes normalized to the control group. EB volume is presented in % of the respective control. Thirty-two EBs per condition, mean, and SD for three biological replicates of each cell line (BIONi010-C valproic acid *n* = 2). Stars indicate significant differences of respective concentrations vs. control calculated with an ordinary one-way ANOVA on means of 32 EBs per condition

We noticed that not only the beat score changed in response to treatments but also the size of the contracting spheres (this can also be seen on suppl. videos 1–5). Therefore, we investigated the size of the spheres on microscopy images taken on the last day of the assay after scoring of the beating. This analysis showed that thalidomide treatment led to the same trend in decreasing EB volume as in decreasing the beat score (Fig. 5). However, the decrease in volume was stronger than the decrease in beat score, as the volume was already decreased by almost 60% at the lowest concentration. Moreover, the effect was statistically significant in the IMR90-4 cell line, which was not seen with the beat score. Treatment with VPA did not lead to apparent effects in the BIONi010-C cell line but decreased the EB volume at the highest concentrations in IMR90-1 and IMR90-4 (Fig. 5). Epoxiconazole treatment gave rise to significantly smaller spheres in the BIONi010-C and IMR90-4 cell lines at all concentrations, while only a non-significant trend was seen in IMR90-1 (Fig. 5). This is in contrast to the analysis of the beat score, where IMR90-1 showed a decreased beat score already at 1.3 µM epoxiconazole (Fig. 4).

## Discussion

In this study, we developed and characterized a novel assay for predicting chemically induced developmental toxicity, which we term the PluriBeat assay. This assay has several advantages over previously published assays as it is fast to conduct in only 8 days using 96-well plates, which also bears automation potential. In our hands, the assay was more robust than the embryonic stem cell test (EST) with mouse ESCs and it was reproducible between several biological replicates. Most importantly, the PluriBeat assays are based on human pluripotent stem cells cultured in 3D as EBs, making it very relevant for the human embryo.

### Testing thalidomide, valproic acid, and epoxiconazole in the PluriBeat assay

We used thalidomide as a first reference compound in our assay, because it is a known teratogen and has not shown developmental toxicity in murine-based in vitro assays (Uibel et al. 2010). The concentrations of thalidomide used in our study included clinical plasma concentrations for the therapeutic use of thalidomide and are, therefore, relevant for human exposure (1–6 µM) (Chung et al. 2004; Kodama et al. 2009). For comparison, a regular dose for pregnant women of 200 mg thalidomide, as it was prescribed before the adverse effects on developing embryos and fetuses were known, would give rise to an internal exposure of 4.7 µM (Teo et al. 2004). In this direct comparison of in vitro responses with internal therapeutic plasma concentrations, we do not take into consideration that internal exposure of the cells likely differs from nominal in vitro concentrations (e.g., due to evaporation of compound or adhesion to plastics) or any toxicokinetic issues in vivo. However, in spite of that these therapeutic plasma concentrations were at a level where we detected effects on the beat score in vitro. Effective concentrations of thalidomide in in vitro assays for developmental toxicity have been reported at concentrations ranging between 0.5 and 450 µM (Mayshar et al. 2011; Palmer et al. 2013; Aikawa et al. 2014; Kameoka et al. 2014; Aikawa 2020). The response to thalidomide in the PluriBeat assay using BIONi010-C (IC_25_ 2.0 µM) and IMR90-1 (IC_25_ 14.3 µM) was comparable to previously published data. Interestingly, we were not able to detect a significant effect of thalidomide on the beat score with the IMR90-4 cell line but only a trend in decreasing this score. When analyzing the volume of the spheres, we did find a significant decrease at 18 and 36 µM thalidomide. In BIONi010-C and IMR90-1, this was already seen from 2.3 µM. Together, our data on the beat score and sphere volume show that thalidomide has a negative effect on the differentiation of all three cell lines into spheres containing cardiomyocytes. However, the effect is weakest in IMR90-4, and we hypothesize that this cell line is less sensitive to this test compound and stronger effects would be observed at higher concentrations. We only tested concentrations up to 36 µM, while thalidomide did not lead to cytotoxicity up to 200 µM, and the effects of higher concentrations remain to be elucidated in future experiments.

Intriguingly, we did not detect any impact of valproic acid on the beat score at test concentrations up to 300 µM in our assay. Previous in vitro developmental toxicity studies have reported IC_50_ values above this concentration (868–1000 µM) (Aikawa et al. 2014; Shinde et al. 2016), but also below at concentrations ranging from 3 to 205 µM (Uibel et al. 2010; Palmer et al. 2013; Kameoka et al. 2014; Aikawa 2020). The two publications by Aikawa use a similar approach as the PluriBeat assay, but because no data on the quality or reproducibility of the experiments is shown, we cannot determine the reason for this discrepancy. We speculate that we were not able to test high enough concentrations to affect the beat score due to cytotoxicity above 300 µM valproic acid. Of note, the concentrations tested in this study did not reach levels relevant for human exposure either. Therapeutic serum levels of valproic acid are around 700 µM (Dasgupta 2016), which is much higher than the concentrations tested in our assay (300 µM). Also in ex vivo cultures of rat embryos (gestation day 10) toxic effects of valproic acid were only found at 800 µM (Rettie et al. 1986). In contrast to the beat score, we found effects on the EB volumes in IMR90-1 and IMR90-4 at 200 µM and 300 µM valproic acid, respectively. This suggests that valproic acid has an impact on the differentiating cells; however, it did not manifest as a decrease in the beat score. The molecular mechanism of its teratogenicity is not known, but valproic acid leads to neurodevelopmental defects in children such as spina bifida, malformations of the heart such as atrial septal defect, and other urogenital, craniofacial, and digital abnormalities (Jentink et al. 2010). Although in vitro assays have mostly focused on the neurodevelopmental effects of valproic acid, other genes have been found to be deregulated in differentiating human stem cells exposed to valproic acid: *CLDN10*, which is related to heart tube looping; *BCL2*, a key regulator of embryogenesis; and *PRKCB*, which is involved in various cellular processes (Shinde et al. 2016). These studies indicate that valproic acid leads to effects in assays based on general cellular and developmental processes (Uibel et al. 2010; Palmer et al. 2013; Kameoka et al. 2014) or neurodevelopmental effects (Shinde et al. 2016). From this, we conclude that our PluriBeat assay based on the readout of contracting cardiomyocytes might only detect chemicals that interfere with other general developmental pathways or pathways specific to heart development. Chemicals that are specific to neurodevelopmental defects might not be detected in our system, but this remains to be elucidated in future studies with such model compounds.

Epoxiconazole led to a significant effect on the beat score in all three cell lines, while the volume of the spheres was only reduced in the BIONi010-C and IMR90-4 cell lines, not in IMR90-1. This is opposed to the effect which we saw on the beat score, which was markedly reduced in IMR90-1. We speculate whether the effects on the beating and the size of the spheres are mediated through different cellular mechanisms, which should be the objective of future studies. Still, epoxiconazole affected the beat score even more potent than thalidomide, as it decreased the beating at concentrations as low as 1.3 µM (as compared to the LOEC of thalidomide of 2.3 µM) and reduced the proportion of beating cardiac EBs to almost 0%, which was not observed with thalidomide. With this, epoxiconazole was more potent than thalidomide in the PluriBeat assay. We also found a higher potency than in the mouse embryonic stem cell test (mEST) based on mouse ESCs, where we previously reported an IC_50_ of 34 µM (Dreisig et al. 2013). We also have experience from in vivo experiments with epoxiconazole showing feto-toxicity in rats (Taxvig et al. 2007). In conclusion, we found indications that epoxiconazole has the potential to adversely affect human embryos and fetuses at extraordinary high human exposure levels.

Importantly, our results on thalidomide also indicate that our hiPSC-based assay might indeed uncover toxicities of compounds that are specific to human cells and that have not been detected in established assays using rodent cells or models.

### Comparing beat score and EB volume

It was important to investigate a second readout in the PluriBeat assay, which was a measure of the total cell number in the spheres on the last day of the assay. It was obvious that the spheres with a low beat score also had a smaller size. Interestingly, our comparison of the EB volume and beat score correlated well for all cell lines and compounds, except for thalidomide in IMR90-4 and epoxiconazole in IMR90-1, which are discussed in the previous section. We hypothesize that the chemicals might slow down cell proliferation, which leads to a lower cell number and, therefore, smaller volume of the spheres on day 8 of the assay. Eventually, reduced cell proliferation also affects differentiation (Mummery et al. 2012; Liu et al. 2019). We find that the reduced sphere volume strengthens the characterization of the PluriBeat assay and improves the interpretation of the assay outcome.

### Comparison of BIONi010-C, IMR90-1, and IMR90-4

To our knowledge, this study is also the first published in vitro toxicity study using three different hiPSC lines. Our results underline the importance of increasing the number of pluripotent cell lines tested. While the cell lines showed a similar efficiency to differentiate into cardiomyocytes, they exhibited some differences in sensitivity to thalidomide and epoxiconazole exposure. Intriguingly, BIONi010-C was more sensitive towards thalidomide, whereas IMR90-1 was more sensitive towards epoxiconazole. With this, we can exclude that one of the cell lines is generally more susceptible to perturbations by chemicals and hypothesize in accordance with Fossati et al. (2016) that either intrinsic molecular features of the cell lines based on the genetic background of the donor, the type of donor cell, reprogramming factors, or cultivation-related variations are responsible for the different sensitivities. The technique of reprogramming for the derivation of the cell lines did indeed differ between the used cell lines. IMR90-1 and IMR90-4 were derived by the integration of exogenous transcription factors, which are silenced after the reprogramming (Yu et al. 2007), while BIONi010-C do not carry exogenous factors (Rasmussen et al. 2014). Silencing can be inefficient and the transgenes re-activated, leading to problems in differentiation (Hu 2014). Indeed, we found that in IMR90-4, NANOG was not significantly downregulated during cardiomyocytes differentiation (Fig. 3). This might explain the differences between IMR90-4 and the other two cell lines to some extent and we suggest to further explore the underlying molecular mechanisms in future studies. Until then, our findings support the notion to use several hiPSC lines in parallel. We propose using at least two cell lines and propose BIONi010-C and IMR90-1, however, testing with more chemicals will be needed to support this suggestion. We would also like to suggest using a tiered approach, i.e., testing chemicals in one cell line (BIONi010-C) with subsequently testing chemicals with a negative result in a second cell line (IMR90-1).

### Comparison to other developmental toxicity assays

There are other stem-cell-based in vitro assays for developmental toxicity reported to date, of which the mEST has by far been the most frequently used. The EST was first developed with mouse ESCs and later adapted to human ESCs (Spielmann et al. 1997; Adler et al. 2008). The mEST assesses the teratogenic potential by comparing spontaneous outgrowth of beating cardiomyocytes in EBs compared to cytotoxic effects on pluripotent ESCs and fibroblasts. These three endpoints are fed into a mathematical prediction model to classify chemicals into three classes: non, weakly, or strongly embryotoxic (Seiler and Spielmann 2011). The mEST has been validated scientifically (Genschow et al. 2002), but has not become part of a guideline test for regulatory approval of chemicals (Marx-Stoelting et al. 2009). Thus, these are still based on animal experiments (OECD TG 414). The mEST is very labor-intensive and the reproducibility is generally inadequate. Therefore, many subsequent studies have attempted to overcome these limitations and we also considered those for the development of our assay:

First of all, the EST is labor-intensive regarding the differentiation of EBs in hanging drops (Marx-Stoelting et al. 2009). Therefore, we decided to improve the generation of EBs using conical-shaped 96-well plates. We showed that our method gives rise to uniform EBs across one 96-well plate, as the variation in cell number across one plate was as low as 7.4% on day 0 and 8.1% on day 7. With this, we conclude that the use of conical-shaped 96-well plates is a good alternative to hanging drops. Of note, the 96-well plate-based method is easier to conduct in the laboratory and allows for the use of automated liquid handling devices.

Moreover, the original EST uses three endpoints and a mathematical prediction model to classify chemicals into groups according to their hazard towards developing embryos (Spielmann et al. 1997). We decided not to implement a comparable prediction model, but rather to determine the LOEC at a non-cytotoxic concentration for use in hazard assessment, as we envision the PluriBeat assay to become one assay in a larger panel of tests for developmental toxicity.

Furthermore, we observed that the previously suggested classification method into beating or non-beating EBs was not sensitive enough to detect subtle effects on the beating of the EBs (data not shown). We, therefore, developed the beat score ranking, which yields quantifiable results and also takes impaired or partially contracting EBs into consideration. With the LOEC for effects on beat score, we hypothesize to detect more subtle toxicities compared to other assays. Importantly, to reduce subjectivity of the scoring results, we implemented the scoring criteria described in the Methods section, and suggest to use these criteria to minimize differences in outcomes between scorings of different individuals.

The major limitation of the original EST is that it is based on murine and not human cells (Seiler et al. 2011). This could lead to species differences in response to chemical exposure, as teratogens in rats and rabbits show only 70–80% concordance with results in humans (Daston and Knudsen 2010). Therefore, the EST has also been developed with human ESCs using human fibroblasts and human ESCs (Adler et al. 2008). However, a human EST has never been validated. Its major drawback is the ethical concern related to the use of human ESCs, because human embryos are sacrificed for their retrieval (Thomson et al. 1998; Seiler et al. 2011). Therefore, we decided to use hiPSC in the PluriBeat assay, since they possess no ethical concerns and can mimic the developing embryo to the same degree as ESCs. hiPSC has been shown to predict the embryotoxicity of thalidomide using the original EST protocol, but no data revealing the quality of the hiPSC culture and cardiac differentiation or the reproducibility between experiments were shown (Aikawa et al. 2014; Aikawa 2020). Therefore, we cannot compare our assay to this study in terms of quality, but we clearly detected thalidomide toxicity at lower concentrations (IC_25_ 2.0 µM compared to IC_50_ 450 µM in Aikawa et al. 2014 and IC_50_ 5.5 µM in Aikawa 2020).

The most advanced EST based on human cells to date is the human pluripotent stem cell test (hPST), developed by Kameoka et al. (2014). This test is a high-throughput assay based on hESCs to screen for teratogenicity by measuring the nuclear levels of Sox17 (Kameoka et al. 2014). The test takes 3 days and detected thalidomide with an IC_50_ of 0.5 µM. However, this method is based on a stem cell source with ethical limitations namely hESC, and the cells are cultured in monolayer, which is less relevant for the physiological in vivo situation than 3D culture. This might also explain the lower IC_50_ values determined in that study compared to our PluriBeat assay, as compounds can directly affect the cells in monolayer and do not have to penetrate the spheroids. However, we are confident that 3D spheroids are more relevant to the developing human embryo and might, for example, be excellent models to study molecular mechanisms of toxicants such as thalidomide.

It was very important for us to show that our assay mimics the in vivo developing embryo well and to show the relevance for humans. We, therefore, decided to use EBs as they are structurally and functionally similar to the early embryo (Brickman and Serup 2016). During human embryonic development, the heart starts beating on day 21 and is the first functional organ to be developed. Our model is, therefore, a surrogate of the first weeks of development and we showed that key molecular events of heart differentiation occur also in our EBs, as marker genes for mesoderm, cardiac mesoderm, cardiac progenitor cells, as well as cardiomyocytes are expressed during the course of differentiation. We recently also compared the expression of the markers in our model to their expression in the developing heart of a large mammalian model, the pig, and found a significant overlap, while only a few markers differed (Volpini et al. 2020). With this, we are confident that our model is relevant for predicting human embryonic toxicity of chemicals and drugs.

### Conclusion

In conclusion, we developed an advanced in vitro developmental toxicity assay using a 3D human iPSC model that is reproducible, robust, and mimics key molecular events of early human embryonic heart development. A proof of principle was illustrated by testing thalidomide, valproic acid, and epoxiconazole, with the conclusion that the assay indeed can confirm the known developmental toxicity of thalidomide and can detect the developmental toxicity of epoxiconazole previously observed in rodents and the mEST. An adverse effect of valproic acid was only detected with EB volume as a readout, not the beat score, suggesting that the PluriBeat assay might be limited to toxicants that target general developmental pathways or those specific for cardiac development.

In future, more chemicals are to be tested to elucidate the applicability domain of the PluriBeat system and to validate the assay. These chemicals should include positive as well as negative controls to determine the false-positive rate.

We envision that this assay can be part of a panel of in vitro assays together with assays based on other endpoints, for example for neurodevelopmental toxicity, and that such a panel together with computational models can predict human developmental toxicity better than rodent models and might eventually replace in vivo experiments.

## Electronic supplementary material

Below is the link to the electronic supplementary material.

Supplementary file1 (DOCX 232 kb)

Supplementary file2 (MP4 2944 kb)

Supplementary file3 (MP4 5779 kb)

Supplementary file4 (MP4 2778 kb)

Supplementary file5 (MP4 3316 kb)

Supplementary file6 (MP4 1108 kb)
