# Supplementary material for: A novel human pluripotent stem cell-based assay to predict developmental toxicity
Source: Arch Toxicol. 2020 Jul 22;94(11):3831–46. doi: 10.1007/s00204-020-02856-6 (PMC7603451; doi:10.1007/s00204-020-02856-6)
Supplement: Supplementary file 1 — Supplementary file1 (DOCX 232 kb) [file 204_2020_2856_MOESM1_ESM.docx]

**Supplementary table 1**

| **mTeSR-ROCK** | |
| --- | --- |
| **Component** | **Vol.** |
| mTeSR1 (STEMCELL Technologies 85850) | 10ml |
| ROCK (5 mM, Cayman Chemical 10005583) | 20µl |
| PSG (Life technologies 10378016) | 100µl |

| **DO medium** | |
| --- | --- |
| **Component** | **Vol.** |
| KO-DMEM | 10ml |
| PSG (Life technologies 10378016) | 100μl |
| ITS (BD 354351) | 10μl |
| Y (10 mM, Abcam 120129) | 10μl |
| FGF2 (10 μg/ml, Peprotech 100-18B) | 10μl |
| Activin A (10 μg/ml, eBioscience 34-8993-85) | 10μl |
| CHIR (1 mM, Axon Medchem 1386) | 25μl |
| BMP4 (10 μg/ml, R&D 314-BP-010) | 1μl |

| **TS medium** | |
| --- | --- |
| **Component** | **Vol.** |
| KO-DMEM (Life technologies 10829018) | 10ml |
| PSG (Life technologies 10378016) | 100μl |
| TS * | 100μl |
| Asc (250 mM, Sigma-Aldrich 49752) | 10µl |

| **Wnt medium** | |
| --- | --- |
| **Component** | **Vol.** |
| KO-DMEM (Life technologies 10829018) | 10ml |
| PSG (Life technologies 10378016) | 100μl |
| TS * | 100μl |
| Asc (250 mM, Sigma-Aldrich 49752) | 10μl |
| Wnt (2mM, Tocris Cat.-No. 5148) | 20μl |

*TS is prepared by adding 1ml of sodium selenite (27 mg sodium selenite Sigma-Aldrich S5261 in 400 ml PBS w/o Ca and Mg) to the 99 ml transferrin (55 mg transferrin Sigma-Aldrich T8158 in 99 ml PBS w/o Ca and Mg)

**Supplementary table 2**

Taq Man gene expression assays

| OCT4 (POU5F1) | Hs04260367_gH |
| --- | --- |
| NANOG | Hs02387400_g1 |
| SOX2 | Hs01053049_s1 |
| BRACHYURY | Hs00610080_m1 |
| NKX2.5 | Hs00231763_m1 |
| TNNT2 | Hs00943911_m1 |
| MHCA | Hs01101425_m1 |
| MHCB | Hs01110632_m1 |
| Beta Actin | Hs01060665_g1 |
| GAPDH | Hs02786624_g1 |

**
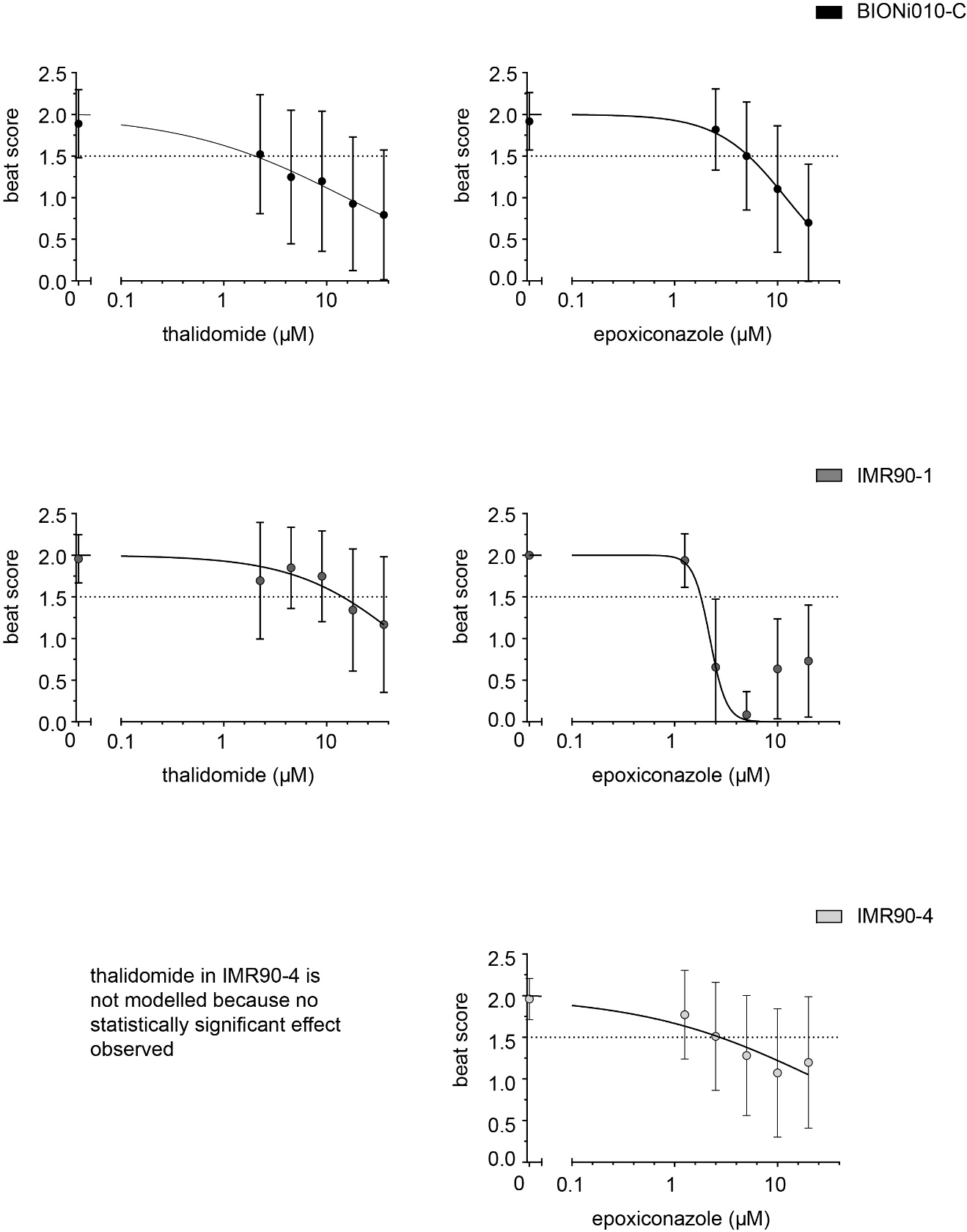
**

**Supplementary figure 1:** Concentration-response curves for thalidomide and epoxiconazole in the three cell lines BIONi010-C, IMR90-1 and IMR90-4. A four parameter curve fit with the lower limit constrained to 0 and upper limit to 2, as well as F = 75 was applied to calculate 25 % inhibition. Graphs represent mean and SD of >90 EBs from 3 independent experiments for each cell line. Dotted lines indicate 25 % inhibition of response.
